# Arbo-Score: A Rapid Score for Early Identification of Patients with Imported Arbovirosis Caused by Dengue, Chikungunya and Zika Virus

**DOI:** 10.3390/microorganisms8111731

**Published:** 2020-11-04

**Authors:** Iacopo Vellere, Filippo Lagi, Michele Spinicci, Antonia Mantella, Elisabetta Mantengoli, Giampaolo Corti, Maria Grazia Colao, Federico Gobbi, Gian Maria Rossolini, Alessandro Bartoloni, Lorenzo Zammarchi

**Affiliations:** 1 Department of Experimental and Clinical Medicine, University of Florence, 50134 Florence, Italy; iacopo.vellere@unifi.it (I.V.); filippo.lagi@unifi.it (F.L.); michele.spinicci@unifi.it (M.S.); antonia.mantella@unifi.it (A.M.); giampaolo.corti@unifi.it (G.C.); gianmaria.rossolini@unifi.it (G.M.R.); alessandro.bartoloni@unifi.it (A.B.); 2 Infectious and Tropical Diseases Unit, Careggi University Hospital, 50134 Florence, Italy; mantengolie@aou-careggi.toscana.it; 3 Referral Centre for Tropical Diseases of Tuscany, Infectious and Tropical Diseases Unit, Careggi University Hospital, 50134 Florence, Italy; 4 Clinical Microbiology and Virology Unit, Careggi University Hospital, 50134 Florence, Italy; colaog@aou-careggi.toscana.it; 5 Department of Infectious/Tropical Diseases and Microbiology, IRCCS Sacro Cuore Don Calabria Hospital, 37024 Negrar, Verona, Italy; federico.gobbi@sacrocuore.it

**Keywords:** dengue, Zika, chikungunya, imported, timing, diagnosis, travelers, score

## Abstract

Background: Chikungunya (CHIKV), Dengue (DENV), and Zika (ZIKV) viruses present significant clinical and epidemiological overlap, making an accurate and rapid diagnosis challenging. Timely activation of preventive vector control measures is crucial to avoid outbreaks in non-endemic settings. Diagnosis is based on combination of serological and molecular assays which could be time consuming and sometimes disappointing. Methods: We report the results of a retrospective case-control study carried out at a tertiary teaching hospital in Italy, including all febrile subjects returning from tropical countries during the period 2014–2019. Controls were travelers with other febrile illnesses who tested negative in laboratory analysis for CHIKV, DENV, ZIKV arbovirosis. A score weighted on the regression coefficients for the independent predictors was generated. Results: Ninety patients were identified: 34 cases (22 DENV, 4 CHIKV, and 8 ZIKV) and 56 controls. According to our results, myalgia, cutaneous rash, absence of respiratory symptoms, leukopenia, and hypertransaminasemia showed the strongest association with arbovirosis. Combining these variables, we generated a scoring model that showed an excellent performance (AUC 0.93). The best cut-off (>=2) presented a sensitivity of 82.35% and specificity of 96.43%. Conclusion: A handy and simple score, based on three clinical data (myalgia, cutaneous rash and absence of respiratory symptoms) and two laboratory results (leukopenia and hypertransaminasemia), provides a useful tool to help diagnose arboviral infections and appropriately activate vector control measures in order to avoid local transmission.

## 1. Introduction 

Chikungunya (CHIKV), Dengue (DENV), and Zika (ZIKV) are three *Aedes*-borne viruses with significant clinical and epidemiological overlap associated with possible serological cross-reactivity between viruses belonging to the same family of *Flavivirus*, such as DENV and ZIKV, making an accurate and rapid diagnosis challenging [1,2,3]. During the last 50 years, many DENV outbreaks occurred, which has contributed to the spread of the virus worldwide [4]. Circulation of CHIKV was limited to the African and Asian regions until 2013, when it reached the Americas [5]. In 2007, the first ZIKV outbreak outside Asia and Africa occurred in the Yap State, Micronesia [6], and later, in 2015, the virus spread to Latin America [7].

These diseases have become a public health concern also in non-tropical countries due to an increasing number of imported infections [8]. The worldwide diffusion of competent vectors such as *Aedes aegypti* and *Aedes albopictus* allowed autochthonous transmission reaching the southern United States [9] and the Mediterranean basin [10,11,12]. Even though imported cases of ZIKV have decreased in the last two years [13], in 2019 autochthonous transmission was still registered in southern France [14]. Additionally, human-to-human modes of transmission are possible even in the absence of vectors, and concerns about transmission via blood transfusions and sexual intercourse are increasing [15,16,17]. 

Even though the initial clinical manifestations may be similar for the three arboviral infections, as well as for other febrile diseases commonly affecting travelers [18], the complications of these three infections are very different and specific and require targeted medical interventions. DENV could rapidly turn into a life-threatening shock and hemorrhagic syndrome. In this case, non-steroidal anti-inflammatory drugs and invasive procedures are contraindicated, and proper oral or intravenous hydration is the mainstay of treatment. ZIKV represents a concern because of sexual and vertical transmission, with the risk of microcephaly and other congenital malformations, but also with increased risk of Guillain-Barré syndrome [19]. Affected individuals have to be individually counseled to avoid sexual transmission, and affected pregnant women have to be advised about the possible fetal risk and appropriately monitored. CHIKV produces arthralgia often lasting for years, which requires anti-inflammatory, immunomodulatory or, sometimes, immunosuppressive treatment [20]. In the context of travel medicine, especially in countries such as Italy where competent vectors are widespread, a rapid diagnosis is essential to diagnose patients with *Aedes*-transmitted arbovirosis in order to perform preventive measures of vector elimination, with the aim of preventing the emergence of autochthonous foci [21,22,23].

Regarding diagnosis, during the first five to seven days from the onset of symptoms, the gold standard is represented by nucleic acid amplification tests (NAAT) to detect viral RNA. In the case of DENV, detection of non-structural protein 1 (NS1) is also possible either with enzyme-linked immunosorbent assay (ELISA) or rapid immunochromatographic test (ICT). After one week from the onset of symptoms, diagnosis relies on serodiagnostic tests, with seroconversion of IgG or at least four-fold increase of IgG titers using ELISA, indirect fluorescent antibody test (IFAT) or plaque reduction and neutralization test (PNRT) [24,25,26]. Rapid diagnostic tests are commercially available for these three infections. However, the performance of these tests is not the same for these three pathogens. For DENV, several rapid commercial tests with excellent performance are available to detect both the NS1 antigen (in the first 7 days of symptoms) and antibodies (after 5 days from symptoms onset), usually ensuring a rapid and reliable diagnosis, however, for CHIKV and ZIKV the reported performance of rapid commercial tests is often disappointing [27,28,29]. Recently, a rapid Loop-Mediated Isothermal Amplification Technique based test (RT-LAMP) has been described to detect ZIKV-RNA and DENV-RNA with high sensitivity [30,31].

The main aim of this study was to develop a score for the early identification of ZIKV, CHIKV and DENV imported arbovirus infections, based on anamnestic and laboratory findings in febrile ill travelers returning from an endemic country, which could be predictive for each of the three arbovirosis. Secondly, this study aims to find distinctive characteristics to differentiate each of the three arbovirosis described.

## 2. Materials and Methods 

### 2.1. Study Design, Setting and Inclusion Criteria

This was a retrospective, unmatched case-control study.

The study was carried out at the Infectious and Tropical Disease Unit of Careggi Hospital, a tertiary teaching hospital in Florence, Italy, in the period from 1 January 2014, to 31 December 2019. 

We included all febrile subjects returning from the tropics referred to our outpatient or inpatient service (depending on the severity of their symptoms and comorbidities) for suspected imported arbovirosis. The inclusion criteria were as follows: 

Fever at the time of the medical visit, or history of fever (measured axillary temperature above 37.5 °C) developed within 2 weeks after returning from a tropical or subtropical country endemic for at least one of the three arboviruses (DENV, and/or CHIKV, and/or ZIKV).

(1) presentation to the service no later than 2 weeks from the first day of fever
(2) being tested for DENV, and/or CHIKV, and/or ZIKV

We excluded patients diagnosed with malaria, as the disease was usually ruled out at the beginning of the diagnostic process in travelers returning from malarial areas through blood smear and/or molecular tests. The study was conducted under the provisions of the Declaration of Helsinki and in accordance with the International Conference on Harmonization Consolidated Guideline on Good Clinical Practice. 

### 2.2. Definitions

To identify DENV, CHIKV, and ZIKV infection cases, we used the case definition proposed by the Italian Ministry of Health arbovirosis surveillance plan, published in 2020 [32]. According to these definitions, we included in the “case group” in our study only probable or confirmed cases. Detailed information about these definitions is reported in Supplementary Table S1.

In all febrile subjects returning from the tropics, molecular tests on serum and/or urinary and/or saliva samples were performed in case of the onset of symptoms from less than 7 days. Otherwise, serological tests (IgG and IgM) were performed. In the case of DENV, we also performed the antigen NS1 test within 7 days from the beginning of clinical symptoms. Detailed information about tests performed at our center is reported in Supplementary Table S2.

Controls were travelers with other febrile illnesses (OFIs) who tested negative in laboratory analysis for all three arboviruses. 

For both cases and controls, main epidemiological and clinical data and laboratory values were collected in a database. A tourist was defined as any person who had crossed an international border to travel outside the country where they had settled; if the length of travel lasts more than 6 months, they are called expatriates. A migrant was defined as any person arriving in a country different from their own to settle in the new country. A traveler who had returned to their country of origin to visit relatives and/or friends was defined as a VRF. 

We defined thrombocytopenia as below 140,000/µL, leukopenia below 4000/µL and hypertransaminasemia as alanine aminotransferase (ALT) > 60 UI/mL. C reactive protein (CRP) was defined as normal if below 9 mg/L.

### 2.3. Variables and Statistical Approach

We also collected clinical data regarding the most relevant presented signs and symptoms, as well as laboratory test results including counts of leukocytes, neutrophils and platelets, prothrombin time (PT), ALT and CRP values. Statistical analysis was made using STATA software (v. 14.0). 

Continuous variables were summarized as medians and interquartile ranges (IQRs) and were compared using Mann–Whitney U tests for two-group comparisons. Categorical variables were expressed as frequencies and percentages and were analyzed using Chi-squared or Fisher’s exact tests, as appropriate. Leukocytes, platelets, and ALT values were also interpreted as categorical variables using the reference values available in our laboratory. A univariate logistic regression analysis was performed. Any variable with a *p*-value equal or inferior to 0.2 was considered potentially significant and was further analyzed in multivariate logistic regression (CRP and neutrophil count were excluded because they were available only for limited number of observations). Therefore, we created a simplified score based on the regression coefficients for the independent predictors. Specifically, as previously reported, we divided the smallest regression coefficient by the lowest factor in the model and rounded this quotient to the nearest whole number [33]. We assessed the performance of the score model with the area under the curve (AUC) of receiver operating characteristic (ROC) curve [34]. Therefore, we found the best cut-off by calculating the Youden Index. 

## 3. Results

Detailed information about the selection process of cases and controls is described in the flow chart in Supplementary Figure S1.

The characteristics of controls and cases are summarized in Table 1. 

Overall, 90 patients were identified from a database: 34 cases and 56 controls. Among the cases, 22 were diagnosed with DENV infection (20 confirmed and 2 probable cases), 8 with ZIKV infection (all confirmed), and 4 with CHIKV infection (all confirmed). Diagnostic results are listed in Supplementary Table S3. Fourteen cases (41.2%) and 24 controls (42.9%) were hospitalized. No patients included in the study had dual or triple co-infections. Among cases, the majority were females (*n* = 21, 61.8%), and the median age was 38.5 years (IQR 33–48). Among controls, we had 31 males (55.4%), and the median age was 33 years (IQR 28–47). Italy was the most represented country of birth among cases (*n* = 28) and controls (*n* = 47). Tourists were the majority of controls (*n* = 49) and cases (*n* = 31). We did not find any expatriates among cases and controls. Most cases came from Central America (*n* = 12, 35.3%), whereas most patients with OFIs came from Southeast Asia (*n* = 21, 37.5%). We had a single case coming from Africa (Seychelles). Among cases, the most common presented signs and symptoms were a cutaneous rash (*n* = 23, 67.6%) and myalgia (*n* = 21, 61.8%). Regarding controls, they most commonly presented respiratory symptoms (*n* = 26, 46.4%) or gastrointestinal problems (*n* = 19, 33.9%). Concerning laboratory results, case-patients most commonly presented thrombocytopenia (*n* = 13, 38.2%) and leukopenia (*n* = 20, 58.8%). Hypertransaminasemia was found in 12 cases (35.3%). Returning from Africa resulted in a negative association with ZIKV, CHIKV and DENV infections, although this association was statistically weak.

In Table 2, we report the characteristics of different arbovirosis. 

Regarding DENV infection, myalgia (*n* = 17, 77.3%) was the most common symptom, and leukopenia (*n* = 17, 77.3%) was the most common laboratory finding. Interestingly, there was only one case of severe dengue, which also presented leukocytosis (29,800/µL). Arthralgia and arthritis were the most common clinical features present in CHIKV infections, in 100% and 75% of patients, respectively. The most frequent symptoms of ZIKV patients were rash (100%) and headache (75%). Regarding the number of neutrophils, the median value resulted smaller in DENV infection (1418/µL). CRP was positive in 8 of 24 tested cases (33.3%): the highest value (189 mg/dL) was registered in the case of severe dengue, where no bacterial superinfection was demonstrated. Among cases with hypertransaminasemia, 11 (91.7%) were DENV infections; there were no ZIKV patients presenting with ALT elevation. Median platelet level was higher in CHIKV (349 × 10^3^/mcL) than DENV (142 × 10^3^/mcL) and ZIKV (158 × 10^3^/mcL). According to our multivariate analysis, myalgia, rash, absence of respiratory symptoms, leukopenia and hypertransaminasemia showed the strongest association with arbovirosis (Table 3). 

**Figure 1:**
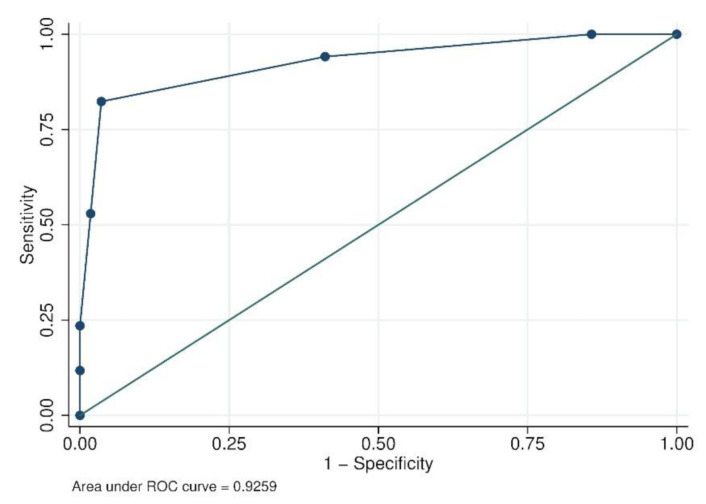
Receiver operating characteristics (ROC) curve for the score model with leukopenia, hypertransaminasemia, myalgia, rash, respiratory symptoms (*b/w*).

Therefore, using regression coefficients, we generated a scoring model including +2 points for leukopenia, +1 point for hypertransaminasemia, +1 point for rash, and +1 point for myalgia and −1 point for respiratory symptoms. The receiver operating characteristics (ROC) curve showed an AUC of 0.93 (Figure 1).

The best cut-off point resulted in greater than or equal to 2 (Table 4).

## 4. Discussion

Physicians who manage febrile, returning travelers must always place priority on the differential diagnosis of conditions that are treatable, that may cause serious sequelae or death, and pose a risk to public health [35]. Arbovirosis fulfills all the aforementioned priority conditions. A subject returning from an endemic area represents a risk for the emergence of autochthonous cases in areas where competent vectors are present; hence, an appropriate diagnostic approach is crucial to limit this risk. Very recently, small clusters of autochthonous DENV infection have been reported in northeast Italy and southern France, highlighting that, despite current travel limitations imposed by the COVID-19 pandemic, imported arbovirosis may still represent a challenge for clinicians and public health officers in temperate regions [36,37,38,39]. In 2016, researchers from Lausanne Infectious Disease Unit proposed one diagnostic algorithm for travelers with nonspecific febrile illnesses returning from regions experiencing simultaneous outbreaks of DENV, CHIKV, and ZIKV infections, based on serology results [40]. In our opinion, this approach could present some limitations. Firstly, serology in the first week from the onset of symptoms could be negative. Secondly, NAAT and serological ELISA tests could be available only in referral centers [41]. Therefore, patients who have been previously vaccinated for another *Flavivirus* such as Yellow Fever Virus, Japanese Encephalitis Virus, and Tick-borne Encephalitis Virus, could present false positive serology for DENV or ZIKV. Considering that the cost of routine surveillance preventive measures, such as comprehensive larviciding, over whole urban areas could overcome health benefits, especially in larger municipalities, rapid identification of imported arboviral infections is crucial to rapidly activate preventive measures localized in a specific area to avoid local transmission [42]. Regarding DENV infection, another aspect to consider is the variability of the viremic period, which could last on average nine days in travelers after the onset of symptoms, compared to seven days in endemic settings [43]. At the same time, the extrinsic incubation period (EIP) in competent vector mosquitos, generally referenced to be 8–12 days, could be more variable and inversely correlated with temperature [44]. In a study published in 2019, lower leukocyte and platelet levels resulted in significant associations with DENV, CHIKV, and ZIKV in the differential diagnosis of imported fever [45].

According to our results, leukopenia, cutaneous rash, hypertransaminasemia, respiratory symptoms (at least one among cough, respiratory distress, sore throat and rhinorrhea) and myalgia were the best parameters to predict arbovirosis in a febrile traveler returning from a tropical country. A simple score, called ARBO-SCORE, based on three clinical data values v(myalgia, cutaneous rash and absence of respiratory symptoms) and two easily obtainable laboratory results (leukopenia and hypertransaminasemia), has been shown to provide a useful tool to help diagnose arboviral infections and to effectively activate vector control measures in order to avoid local transmission, with an accuracy of 91%. The score we generated is straightforward and applicable in peripheral centers. Using our best cut-off point, we obtained a sensitivity of 82.35% and specificity of 96.43%. Regarding different arbovirosis, it is important to emphasize that our case of severe dengue presented leucocitosis, which is a parameter prognostic for this disease [46]. Therefore, there was a notably different median for the platelet count: in a previous study, a lower platelet level was considered to be a prognostic value for DENV versus CHIKV [47]. The differences among these three arbovirosis reflect distinctive characteristics already previously described [48]. The study has several limitations. We excluded many patients because of a lack of information about clinical history or laboratory data. Selection biases could have been additional limitations in this study design. Cases and controls were not matched, so sex and age distribution were different in the two groups. Most cases were DENV infections (64.7%). Presence or history of fever could have limited the number of ZIKV cases. In fact, we excluded three afebrile ZIKV cases. We did not perform any statistical analysis to differentiate among the three arbovirosis, because we estimated too small a number of cases.

## 5. Conclusions 

We elaborated a predictive score named ARBO-SCORE based on three clinical signs (myalgia, rash and respiratory symptoms) and two laboratory values (leukopenia and hypertransaminasemia). The score showed an excellent performance in our population (AUC 0.93), but another validation cohort is necessary to confirm its predictive value. This score could be performed as soon as suspected febrile travelers are encountered, due to its straightforward nature. Therefore, the score is not meant to replace traditional tests for diagnosis, but could help to appropriately activate surveillance systems and perform preventive measures against vectors to avoid autochthonous cases. Further studies are necessary to find clinical predictive scores for each of these arbovirosis.

## Figures and Tables

**Table 1:** Clinical and demographic characteristics of cases: Chikungunya (CHIKV), Dengue (DENV), and Zika (ZIKV) infection cases, and controls (OFIs).

	Control *N* = 56 (%)	Case *N* = 34 (%)	*p*-Value
Gender			0.115
Male	31 (55.4)	13 (38.2)	
Female	25 (44.6)	21 (61.8)	
Age in years			0.307
15–29	22 (39.3)	8 (23.5)	
30–49	21 (37.5)	16 (47.1)	
50–70	13 (23.2)	10 (29.4)	
Region of Birth			0.156
Europe	47 (83.9)	28 (82.3)	
Sub-Saharan Africa	4 (7.1)	0	
Middle South-Asia	1 (1.8)	2 (5.9)	
South-East Asia	1 (1.8)	0	
South America	1 (1.8)	3 (8.8)	
North America	2 (3.6)	0	
Central America	0	1 (2.9)	
Cause of travel			0.856
Tourist	49 (87.5)	31 (91.2)	
Migrant	2 (3.6)	1 (2.9)	
VRF	5 (8.9)	2 (5.9)	
Days between return and onset of symptoms			
(median, IQR)	1 (0–4)	1 (0–4)	0.426
Length of journey in days §	15 (10–21.5)	15 (11–21)	0.896
Days between onset of symptoms and first medical visit			
(median, IQR)	4 (2–7)	4.5 (3–7)	0.230
Returning Continent			0.024
Sub-Saharan Africa	13 (23.2)	1 (2.9)	
North Africa	1 (1.8)	0	
Middle East	1 (1.8)	0	
Middle-South Asia	8 (14.3)	4 (11.8)	
Southeast Asia	21 (37.5)	10 (29.4)	
South America	6 (10.7)	6 (17.6)	
Central America	6 (10.7)	12 (35.3)	
Oceania	0	1 (2.9)	
People returning from Africa	14 (25.0)	1 (2.9)	0.006
Myalgia	17 (30.4)	21 (61.8)	0.003
Rachialgia	14 (25.0)	13 (38.2)	0.184
Headache	28 (50.0)	17 (50.0)	1.000
Retro-orbital pain	6 (10.7)	12 (35.3)	0.005
Conjunctival hyperaemia	2 (3.6)	7 (20.6)	0.009
Gastrointestinal symptoms *	19 (33.9)	11 (32.3)	0.878
Respiratory symptoms **	26 (46.4)	6 (17.6)	0.006
Disgeusia	0	5 (14.7)	0.003
Rash	8 (14.3)	23 (67.6)	0.000
Arthritis	0	4 (11.8)	0.009
Arthralgia	13 (23.2)	10 (29.4)	0.513
Leukocytes/mcL, median (IQR)	6365 (4925–9310)	3725 (2360–5340)	0.000
Leukopenia < 4000/mcL	4 (7.1)	20 (58.8)	0.000
Neutrophil count §§ median (IQR)	3710 (2850–6310) §	2146.5 (1245–3360) §	0.000
Platelets × 10^3^/mcL, median (IQR)	187 (148–236.5)	145.5 (108–183)	0.010
Thrombocytopenia < 140.000/mcL	11 (19.6)	13 (38.2)	0.053
ALT > 60 U/L	11 (19.6)	12 (35.3)	0.099
ALT (U/L) median (IQR)	33.5 (24–49)	34.5 (23–91)	0.516
CRP > 9 mg/L §§§	34 (70.8) §§	8 (33.3) §§	0.002

Footnotes: VRF, visiting relatives and friends; IQR, interquartile range; ALT, alanine transaminase; CRP, C-reactive protein. * At least one of the following: nausea, vomiting, diarrhea. ** At least one the following: coughing, respiratory distress, sore throat, rinorrhea. § data on 82 observations (30 cases and 52 controls) §§ Data on 83 observations (32 cases and 51 controls). §§§ Data on 72 observations (24 cases and 48 controls).

**Table 2:** Clinical and demographic characteristics of DENV, CHIKV and ZIKV arbovirosis.

	DENV *N* = 22 (%)	CHIKV *N* = 4 (%)	ZIKV *N* = 8 (%)
Gender			
Male	9 (40.9)	1 (25.0)	3 (37.5)
Female	13 (59.1)	3 (75.0)	5 (62.5)
Age in years			
15–29	5 (22.7)	0	3 (37.5)
30–49	10 (45.4)	2 (50.0)	4 (50.0)
50–70	7 (31.8)	2 (50.0)	1 (12.5)
Continent of Birth			
Europe	19 (86.4)	1 (25.0)	8 (100.0)
Middle-South Asia	2 (9.1)	0	0
South America	0	3 (75.0)	0
Central America	1 (4.5)	0	0
Returning continent			
Sub-Saharan Africa	1 (4.5)	0	0
Middle-South Asia	4 (18.2)	0	0
Southeast Asia	9 (40.9)	0	1 (12.5)
South America	2 (9.1)	2 (50.0)	2 (25.0)
Central America	6 (27.3)	2 (50.0)	4 (50.0)
Oceania	0	0	1 (12.5)
Myalgia	17 (77.3)	0	4 (50.0)
Rachialgia	10 (45.4)	1 (25.0)	2 (25.0)
Retro-orbital pain	8 (36.4)	0	4 (50.0)
Conjunctival hyperaemia	1 (4.5)	2 (50.0)	4 (50.0)
Gastrointestinal symptoms *	9 (40.9)	2 (50.0)	0
Respiratory symptoms **	4 (18.2)	1 (25.0)	1 (12.5)
Disgeusia	5 (22.7)	0	0
Rash	11 (50.0)	4 (100.0)	8 (100.0)
Arthritis	0	3 (75.0)	1 (12.5)
Arthralgia	3 (13.6)	4 (100)	3 (37.5)
Leukocytes/mcL median (IQR)	3090 (2120–3910)	5240 (3645–6590)	4450 (3985–7195)
Leukopenia < 4000/mcL	17 (77.3)	1 (25.0)	2 (25.0)
Neutrophil count § median [IQR]	1418 (965–2700) §	2970 (1975–3755) §	2540 (2146–4194) §
Thrombocytopenia < 140.000/mcL	10 (45.4)	0	3 (37.5)
Platelets × 10^3^/mcL median (IQR)	142 (88–169)	349.5 (278–414.5)	158 (137–175.5)
ALT > 60 U/L	11 (50.0)	1 (25.0)	0
ALT (U/L) median [IQR]	60 (25–105)	40 (19.5–65.5)	21 (15.5–31)
CRP >9 mg/L §§	5 (29.4) §§	2 (66.7) §§	1 (25.0)

Footnotes: DENV, Dengue virus; CHIKV, Chikungunya virus; ZIKV, Zika virus; ALT, alanine transaminase; CRP, C-reactive protein. * We considered gastrointestinal symptoms to be the presence of at least one of the following: nausea, vomiting, diarrhea. ** We considered respiratory symptoms the presence of at least one the following: coughing, respiratory distress, sore throat, rhinorrhea. § Data on 32 observations (20 DENV, 4 CHIKV, 8 ZIKV). §§ Data on 24 observations (17 DENV, 3 CHIKV, 4 ZIKV).

**Table 3:** Multivariable model and risk score for arbovirosis.

Variables	OR_a_ (95% CI)	*p*	Regression Coefficient	Risk Score Weight
Rash	23.46 (2.79–196.88)	0.004	3.15	1
Thrombocytopenia	0.47 (0.06–3.55)	0.463	−0.76	na
Leukopenia	54.93 (4.56–661.57)	0.002	4.01	2
Hypertransaminasemia	9.41 (1.23–71.66)	0.031	2.24	1
People returning from Africa	0.04 (0.00–12.18)	0.278	−3.10	na
Retro-orbital pain	2.82 (0.35–22.90)	0.331	1.04	na
Conjunctival hyperemia	0.80 (0.07–9.52)	0.862	−0.22	na
Myalgia	13.48 (1.97–92.17)	0.008	2.60	1
Respiratory symptoms	0.10 (0.01–0.74)	0.024	−2.26	−1

Footnotes: na, not applicable.

**Table 4:** Youden index, sensitivity, and specificity of different cut-off points of a score for the early identification of imported arbovirosis infections in febrile ill travelers returning from an endemic country, based on three clinical signs (myalgia +1 point, rash +1 point, respiratory symptoms −1 point) and two laboratory values (leukopenia +2 points, hypertransaminasemia +1 point).

Cut-off Point	Sensibility (%)	Specificity (%)	Youden Index
≥−1	100.00	0.00	0
≥0	100.00	14.29	0.14
≥1	94.12	58.93	0.53
≥2	82.35	96.43	0.79
≥3	52.94	98.21	0.51
≥4	23.53	100.00	0.23
≥5	11.76	100.00	0.12
>5	0	100.00	0

## Supplementary Materials

The following are available online at https://www.mdpi.com/2076-2607/8/11/1731/s1: Figure S1: Flow chart of the selection process of cases and controls; Table S1: Definition of possible, probable and confirmed CHIK, DENV and ZIKV cases according to the 2020–2025 Italian National arbovirosis prevention and surveillance plan; Table S2: DENV, CHIKV, ZIKV serological test performed at Careggi University Hospital, Florence, Italy; Table S3: Diagnostic results of cases.
