# Supplementary figures and images for: Arbo-Score: A Rapid Score for Early Identification of Patients with Imported Arbovirosis Caused by Dengue, Chikungunya and Zika Virus
Source: Microorganisms. 2020 Nov 4;8(11):1731. doi: 10.3390/microorganisms8111731 (PMC7716211; doi:10.3390/microorganisms8111731)

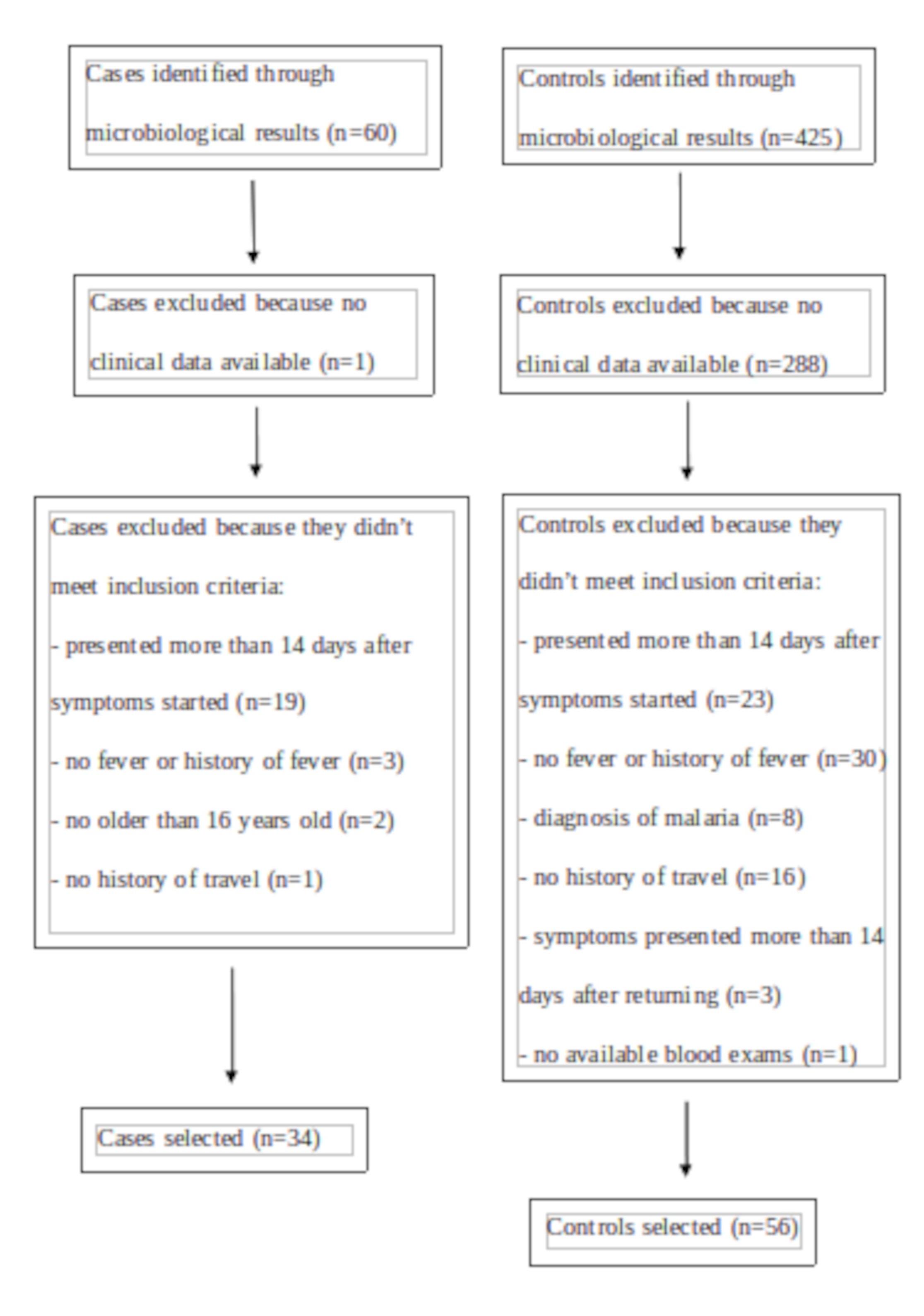

Supplement: Supplementary file 1 [file microorganisms-08-01731-s001.zip › Supplementary data/SF1 definitive.jpg]
