# Supplementary material for: Arbo-Score: A Rapid Score for Early Identification of Patients with Imported Arbovirosis Caused by Dengue, Chikungunya and Zika Virus
Source: Microorganisms. 2020 Nov 4;8(11):1731. doi: 10.3390/microorganisms8111731 (PMC7716211; doi:10.3390/microorganisms8111731)
Supplement: Supplementary file 1 [file microorganisms-08-01731-s001.zip › Supplementary data/Supplementary data definitive.doc]

**Supplementary Table S1.** Definition of possible, probable and confirmed CHIK, DENV and ZIKV cases according to the 2020-2025 Italian National Arbovirosis Prevention and Surveillance Plan.

|  | **CHIKV** | **DENV** | **ZIKV** |
| --- | --- | --- | --- |
| Clinical criteria | Acute fever and polyarthralgia (limiting daily activities) and no other possible causes | Classic Dengue fever. Everybody who has been fever and at least two of the following symptoms:  -nausea  -vomiting  -ocular or retro-orbital pain  -headache  -maculo-papular cutaneous rash  - myalgia  - arthralgia  Predictive signs of severe Dengue:  -abdominal pain or tenderness  -persistent vomiting  -fluid overload  -mucosal bleeding  -hepatomegaly  -restlessness  Severe Dengue:  Dengue with one of the following symptoms:  -severe plasma leakage leading to shock or fluid overload with breathing difficulties  -severe bleeding  -severe organ insufficiency like elevated transaminases >=1000UI/L, altered mental state or heart failure | Cutaneous rash with or without fever and at least one of the following symptoms :  arthralgia  myalgia  non purulent/purulent conjunctivitis |
| Criteria from microbiological results | Criteria for probable case:  CHIKV IgM in one sample of serum  Criteria for confirmed case: (at least one of the following):  - viral isolation in biological samples taken within 7 days from the onset of symptoms  - nucleic acid identification in biological samples  - seroconversion or a four-fold increase of CHIKV antibody title in consecutive samples (taken at least at a distance of 14 days)  - CHIKV IgM in one serum sample and confirmed with neutralization test | Criteria for probable case  DENV IgM in one sample of serum  Criteria for confirmed case (at least one of the following):  - viral isolation in biological samples taken within 7 days from the onset of symptoms  - viral antigen identification in biological samples  - nucleic acid identification in biological samples  - seroconversion or a four-fold increase of DENV antibody title in consecutive samples (taken at least at a distance of 14 days) confirmed with neutralization test  - DENV IgM in one serum sample and confirmed with neutralization test | Criteria for probable case  ZIKV IgM in one sample of serum  Criteria for confirmed case (at least one of the following):  - viral isolation in biological samples  - viral antigen identification in biological samples  - nucleic acid identification in biological samples  -seroconversion or a four-fold increase of ZIKV antibody title in consecutive samples (taken at least at a distance of 14 days) confirmed with neutralization test  - ZIKV IgM in one or more serum sample and confirmed with neutralization test |
| Epidemiological criteria | History of travel or residence in an area with documented transmission of CHIKV in the two weeks before presentation | History of travel or residence in an area with documented transmission of DENV in the two weeks before presentation | History of travel or residence in an area with documented transmission of DENV in the two weeks before presentation  Sexual intercourse with a confirmed case of ZIKV in the previous three weeks (men) or two weeks (women) Sexual intercourse with a person with an history of travel or residence in an area with documented transmission of DENV in the previous three weeks (men) or two weeks (women) |
| Possible case | Everybody who meets clinical and epidemiological criteria | Everybody who meets clinical and epidemiological criteria | Everybody who meets clinical and epidemiological criteria |
| Probable case | Every possible case who meets microbiological criteria for probable case | Every possible case who meets microbiological criteria for probable case | Every possible case who meets microbiological criteria for probable case |
| Confirmed case | Everyone who meets at least one of the microbiological criteria for confirmed case | Everyone who meets at least one of the microbiological criteria for confirmed case | Everyone who meets microbiological criteria for confirmed case |

**Supplementary Table S2.** DENV, CHIKV, ZIKV serological test performed at Careggi University hospital, Florence, Italy.

|  | **Antigen** | **Rapid Diagnostic**  **Test (RDT)** | **Serology** | **PCR** |
| --- | --- | --- | --- | --- |
| Dengue | Dengue NS1 Antigen  Accudiag TM ELISA USA | Onsite Dengue Ag-IgG/IgM Rapid Test CTK Biotech. USA | Vircell MICROBIOLOGISTS  Dengue Elisa IgM capture Granada (Spain) | Dengue Virus general-Type Realtime RT-PCR kit. Liferiver/Shanghai ZJ Biotech Co.- China |
| Zika |  |  | EuroImmun Anti-Virus Zika ELISA  Perkin Elmer Company Germany | ZikaVirus Real Time RT-PCR Kit, Liferiver/Shanghai ZJ Biotech Co. -China |
| Chikungunya |  | NADAL Chikungunya IgM test ICT Nal Von Minden Company Germany | EuroImmun Anti-Virus Chikungunya ELISA Perkin Elmer Company Germany | ChikungunyaVirus RT-PCR Kit, Liferiver/Shanghai ZJ Biotech Co. -China |

**Supplementary Table S3.** Diagnostic results of cases .

|  | **DENV antigen** | **IgM** | **IgG** | **PCR** | **PRNT** | **Degree of Diagnosis Certainty** |
| --- | --- | --- | --- | --- | --- | --- |
| DENV |  |  |  |  |  |  |
| 1 b.ir. | p | p | p | np | np | c |
| 2 b. il. | p | np | np | p | np | c |
| 3 c.s. | np | p | p | n | np | pr |
| 4 c.e. | p | b | n | np | np | c |
| 5 c.m. | p | p | p | np | np | c |
| 6 d.w.i. | np | p | p | np | np | pr |
| 7 g.g.a. | p | n | n | p | np | c |
| 8 i.p.b. | p | n | n | np | np | c |
| 9 l.v. | n | p | n | np | p | c |
| 10 l.d. | p | p | n | n | np | c |
| 11. m.p.p.m. | p | p | p | np | np | c |
| 12 m.v. | p | b | p | np | np | c |
| 13 m.m. | p | p | n | np | np | c |
| 14 p.a. | p | p | n | np | np | c |
| 15 p.m. | p | p | n | np | np | c |
| 16 s.g. | p | p | n | n | np | c |
| 17 m.r. | p | n | n | np | np | c |
| 18 e.m. | p | n | n | np | np | c |
| 19 g.l. | p | p | b | np | np | c |
| 20 b e | p | p | p | np | np | c |
| 21. b.g. | p | b | n | np | np | c |
| 22 f.i. | p | p | p | np | np | c |
| CHIKV |  |  |  |  |  |  |
| 1 b.l.c.a. |  | p | n | np | p | c |
| 2 f.g. |  | b | n | np | p | c |
| 3 l.m.m. |  | p | n | np | p | c |
| 4 o.a. |  | b | n | np | p | c |
| ZIKV |  |  |  |  |  |  |
| 1 a.l. |  | p | p | np | p | c |
| 2 b.d. |  | p | p | np | p | c |
| 3 b.a. |  | n | n | np | p | c |
| 4 c.m. |  | n | n | p | np | c |
| 5 f.b. |  | np | np | p | np | c |
| 6 f.s. |  | np | np | p | np | c |
| 7 m.v. |  | p | n | p | np | c |
| 8 n.a. |  | p | p | np | p | c |

Footnotes: p= positive, n= negative, c= confirmed, pr= probable np= not performed b=borderline.
